# Successful sequential treatment with ofatumumab followed by efgartigimod for refractory autoimmune encephalitis with dual anti-NMDAR and anti-GFAP antibody positivity: first case report

**DOI:** 10.3389/fimmu.2025.1640281

**Published:** 2025-08-13

**Authors:** Xin Tu, Xi Liu, Niao Yang, Dong Sun, Bin Mei, Nao Yan

**Affiliations:** ^1^ Department of Neurology, Zhongnan Hospital of Wuhan University, Wuhan, Hubei, China; ^2^ Department of Cardiology, Wuhan University of Science & Technology, Hanyang Hospital, Wuhan, Hubei, China

**Keywords:** autoimmune encephalitis, anti-NMDAR, anti-GFAP, ofatumumab, efgartigimod

## Abstract

Autoimmune encephalitis (AE) is a heterogeneous disorder mediated by autoantibodies targeting neuronal or glial antigens, with anti-NMDAR encephalitis being the most common subtype, while cases with dual antibody positivity remain exceedingly rare. Standard treatment involves stepwise immunotherapy, but refractory cases often require advanced therapies. This study presents the first reported case of dual anti-NMDAR and anti-GFAP antibody-positive refractory AE in a 24-year-old female who failed first-line treatments (steroids, IVIG) and ovarian teratoma resection. During disease progression, innovative sequential therapy with ofatumumab (OFA), a novel anti-CD20 monoclonal antibody, followed by efgartigimod, an FcRn antagonist, was employed to mitigate profound B-cell depletion risks. The patient exhibited significant clinical improvement, with reduced Modified Rankin Scale (mRS) scores from 5 to 1. OFA induced rapid B-cell depletion, while efgartigimod effectively cleared pathogenic IgG, demonstrating synergistic efficacy. Comparative analysis with literature cases highlighted the superiority of this sequential approach in balancing efficacy and safety.

## 1 Introduction

Autoimmune encephalitis (AE) is a heterogeneous spectrum of disorders mediated by autoantibodies targeting neuronal or glial antigens—including surface proteins, ion channels, synaptic proteins, or intracellular proteins. Its etiology remains incompletely elucidated, potentially involving both endogenous and exogenous triggers. AE may associate with paraneoplastic syndromes, viral infections, and emerging evidence suggests genetic susceptibility may contribute to its pathogenesis (1). The most prevalent autoantibodies include those targeting leucine-rich glioma-inactivated 1 (LGI1), N-methyl-D-aspartate (NMDAR) receptors, contactin-associated protein-like 2 (CASPR2), gamma-aminobutyric acid A (GABAA) or B (GABAB) receptors, and myelin oligodendrocyte glycoprotein (MOG) (1). Diagnosis primarily relies on clinical manifestations—including subacute onset of short-term memory loss, altered mental status, psychiatric symptoms, seizures unexplained by known seizure disorders, or new central nervous system findings—combined with ancillary investigations (e.g., MRI, EEG) (2). Cognitive impairment in autoimmune encephalitis (AE) primarily results from the immune-mediated attack on glutamate receptors, which are indispensable for maintaining normal brain functions such as learning and memory. When antibodies bind to ionotropic or metabotropic glutamate receptors, they disrupt glutamatergic signaling and impair normal interneuronal communication. This disruption predominantly affects cognition-related brain regions, ultimately leading to the prevalent manifestation of memory impairment and other cognitive deficits in AE patients (3). Oligoclonal bands (OCBs), significant inflammatory markers in autoimmune encephalitis, are associated with more pronounced cognitive impairment in patients with dual-antibody seropositivity. These patients typically manifest marked deficits in language and attention domains (4).

Antibody-specific encephalitides exhibit distinct clinical profiles. Anti-NMDAR encephalitis is typically clinically identifiable and associated with IgG antibodies targeting the GluN1 subunit in cerebrospinal fluid (CSF). Core manifestations include psychiatric features and seizures, with most cases demonstrating electroencephalographic (EEG) abnormalities despite generally unremarkable early-stage magnetic resonance imaging (MRI) findings (5). This disorder predominantly affects females, with teratomas identified in up to 58% of young female patients (1). Anti-glial fibrillary acidic protein (GFAP) antibodies represent a rare autoantibody class. Autoimmune GFAP astrocytopathy (GFAP-A) was first described in 2016 as a spectrum disorder of central nervous system autoimmunity affecting the brain, meninges, spinal cord, and optic nerves (6). This condition may occur at any age but predominantly affects middle-aged individuals, with no sex predilection. Core clinical manifestations include fever and headache, while characteristic MRI findings typically reveal linear perivascular radial enhancement perpendicular to the lateral ventricles (6). GFAP-IgG is currently recognized as a disease-specific biomarker for autoimmune GFAP astrocytopathy (GFAP-A). Detection of GFAP-IgG in cerebrospinal fluid (CSF) constitutes the diagnostic gold standard, with disease severity showing positive correlation with GFAP-IgG titer levels. The etiology of GFAP astrocytopathy (GFAP-A) remains incompletely understood. Current evidence associates this disorder with infections and neoplasms, with viral infections being the most commonly reported triggers. Documented viral associations include varicella-zoster virus, cytomegalovirus, and rubella virus (7). Half of GFAP-A patients exhibit preceding symptoms such as influenza-like manifestations or unexplained fever. In a large-cohort study of 102 cases by Flanagan et al., tumors were identified in 34% of patients, with 66% of these developing detectable tumors within 2 years during follow-up. Ovarian teratoma represented the most frequent neoplasm (8).

The standard therapeutic approach for autoimmune encephalitis employs a stepwise strategy: when first-line immunotherapy (high-dose corticosteroids, intravenous immunoglobulin [IVIG], and/or plasma exchange) proves ineffective, escalation to second-line immunotherapy is warranted (9). The primary objective of second-line therapy focuses on targeted elimination of pathogenic antibody-producing B cells or blockade of their effector functions, employing strategies such as signal pathway inhibition, complement-dependent cytotoxicity (CDC), and antibody-dependent cellular cytotoxicity (ADCC) to reduce plasma cell populations, thereby alleviating clinical symptoms (10). In recent years, B-cell-targeting monoclonal antibody therapies have been increasingly employed in AE treatment. Among these, rituximab (RTX) has emerged as the most extensively utilized second-line agent, while other monoclonal antibodies remain in preliminary investigational stages (9). Given the clinical limitations of first-line agents, the delayed therapeutic onset of RTX (typically requiring 4–8 weeks) poses significant challenges in complex encephalitis cases with narrow therapeutic windows. Failure to promptly escalate immunotherapy may lead to catastrophic neurological deterioration due to uncontrolled disease progression. Ofatumumab (OFA), a novel fully human monoclonal antibody, demonstrates superior safety and bioactivity profiles compared to RTX (11). The recommended dosing regimen involves subcutaneous administration of 20mg weekly, transitioning to monthly 20mg maintenance doses from the fourth week onward. Efgartigimod directly targets the neonatal Fc receptor (FcRn), selectively reducing serum IgG concentrations through FcRn-mediated catabolism (12). Emerging evidence demonstrates that FcRn-targeted therapy represents an effective treatment strategy for autoimmune disorders (13). The recommended regimen for efgartigimod involves intravenous administration with a weekly dosing schedule.

This study presents the first documented case of refractory autoimmune encephalitis (AE) with dual positivity for anti-NMDAR and anti-GFAP antibodies. The patient demonstrated suboptimal response to standard first-line immunotherapy and early therapeutic oophorectomy for associated teratoma. During disease progression, we implemented an innovative therapeutic strategy: OFA induction therapy followed by timely transition to efgartigimod to mitigate risks associated with profound B-cell depletion, achieving marked clinical improvement.

## 2 Case presentation

### 2.1 Clinical course and diagnostic workup

A 24-year-old female patient initially presented with fever on December 15, 2024, and received antimicrobial therapy at a community hospital. Despite 4 days of treatment, she experienced persistent febrile episodes, prompting referral to the infectious disease department of a county-level hospital. Following completion of diagnostic workup (including complete blood count), empirical antibiotic therapy was continued without clinical improvement, leading to her admission to our tertiary hospital’s infectious disease unit on December 30.Lumbar puncture revealed cerebrospinal fluid (CSF) abnormalities including: pleocytosis (128 nucleated cells/μL), elevated protein (0.61 g/L), and lymphocytic predominance (88% lymphocytes, 12% monocytes) with activated lymphocytes. Metagenomic next-generation sequencing (mNGS) of CSF identified human herpesvirus 7, while PCR testing confirmed EBV DNA positivity. Brain MRI performed on January 1, 2025 revealed diffuse leptomeningeal thickening and enhancement, suggestive of inflammatory or infectious pathology. No significant subcortical lesions or structural abnormalities were identified. Given the clinical diagnosis of viral meningitis, the patient received ganciclovir antiviral therapy and dexamethasone for anti-inflammatory effects, with adjunctive mannitol for intracranial pressure reduction. This regimen resulted in resolution of fever and headache. The patient was discharged on January 3, 2025 for continued antiviral and glucocorticoid management at a local hospital. However, she developed progressive neuropsychiatric deterioration featuring delirium and behavioral disturbances beginning January 6, necessitating readmission to our neurology department on January 10.Upon admission, the patient had an mRS score of 4. Immediate lumbar puncture revealed elevated opening pressure (175 mmH2O) with CSF abnormalities: pleocytosis (58 cells/μL), elevated protein (0.61 g/L), and lymphocytic predominance (90% lymphocytes, 10% monocytes) with activated lymphocytes. Comprehensive autoimmune encephalitis panel (serum/CSF) demonstrated dual anti-NMDAR and anti-GFAP antibody positivity. CSF metagenomic sequencing showed no pathogenic signatures, and HSV/CMV/EBV PCR testing was negative. Follow-up MRI on January 11, 2025 demonstrated diffuse linear enhancement along cerebral sulci and cortical surfaces, with interval improvement in leptomeningeal thickening and enhancement. These findings favor inflammatory or infectious pathology. No subcortical lesions, structural abnormalities, or spinal cord pathology were identified on whole-spine contrast-enhanced imaging. These findings confirmed anti-NMDAR/GFAP antibody-positive autoimmune encephalitis, occurring 29 days post fever onset after multiple hospital transfers.

### 2.2 Clinical course and therapeutic intervention

**Figure 1:**
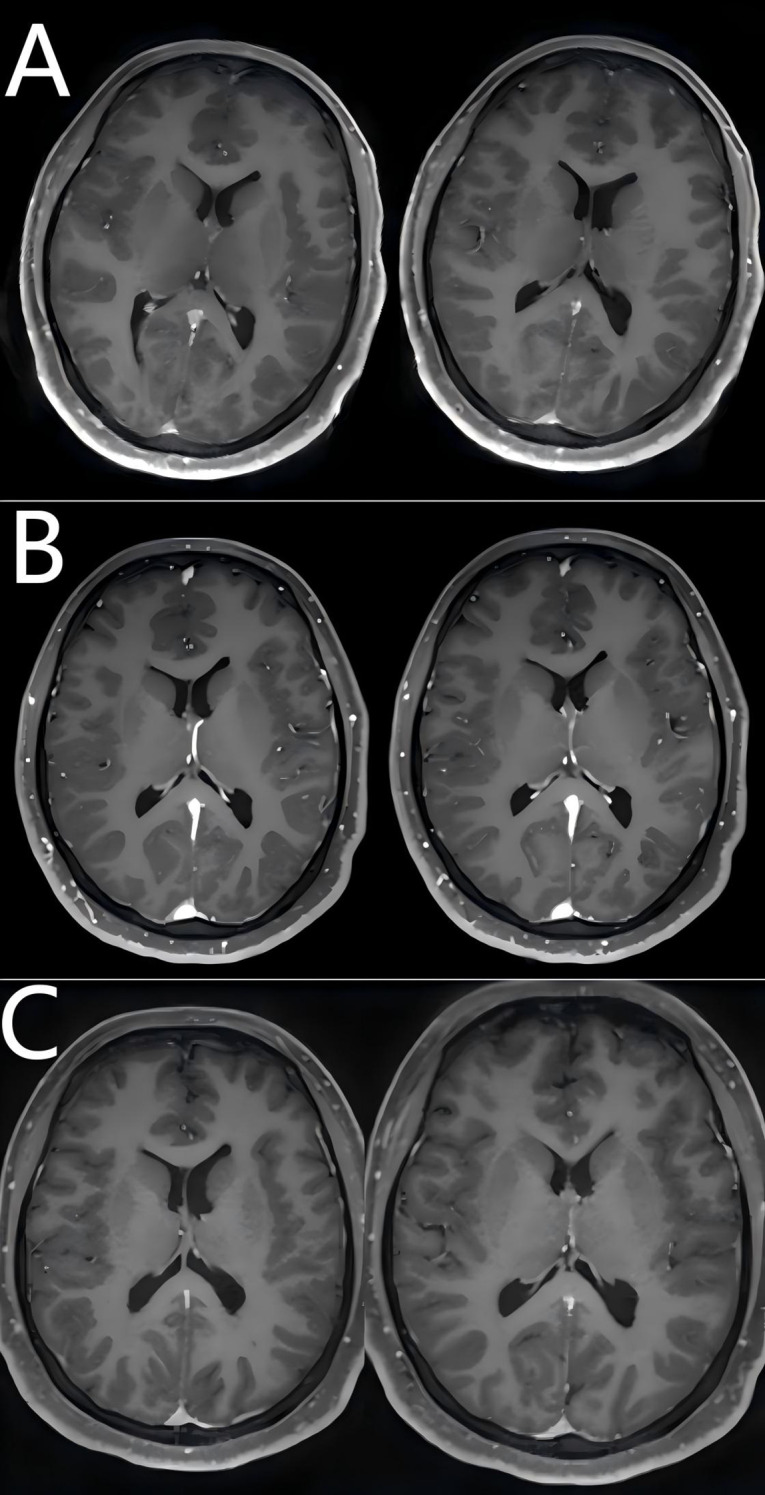
Comparison of pre- and post-treatment brain MRI findings. **(A)** Brain MRI findings at the diagnosis of viral meningitis (January 1, 2025); **(B)** Brain MRI findings at the onset of autoimmune encephalitis (January 11, 2025); **(C)** Brain MRI findings following escalation therapy with first- and second-line agents (March 12, 2025).

Following initial presentation with febrile symptoms, the patient received empiric antimicrobial therapy. On January 12, 2025, laboratory confirmation of concurrent anti-NMDAR and anti-GFAP antibody positivity in both cerebrospinal fluid and serum, coupled with prior positive viral CSF studies, established a diagnosis of post-infectious autoimmune encephalitis secondary to viral meningitis. However, careful review of both serial MRI scans revealed absence of the characteristic perivascular linear radial enhancement perpendicular to the lateral ventricles—the pathognomonic imaging finding in GFAP astrocytopathy. This radiographic deviation may be attributable to relatively lower GFAP-IgG titers compared to NMDAR antibodies and prior glucocorticoid therapy. The diffuse leptomeningeal enhancement observed in this patient primarily stems from autoimmune-mediated meningeal inflammation, characterized by extensive vascular congestion, edema, and perivascular infiltration of plasma cells and lymphocytes. This inflammatory cascade induces endothelial injury with heightened vascular permeability, culminating in blood-brain barrier (BBB) disruption and subsequent contrast enhancement. Electroencephalography (EEG) on January 11, 2025, revealed diffuse low-amplitude 4–5 Hz slow waves. The EEG on January 12, 2025, demonstrated a background rhythm of low-amplitude 13–16 Hz fast activity with dysrhythmia; observable sleep stages were present. Following admission, the patient developed delirium and mania, necessitating heavy sedation. Subsequent EEG monitoring showed diffuse slow waves, with alterations observed upon sedation reduction. No epileptiform discharges were detected. Given the established association between anti-NMDAR encephalitis and teratomas, pelvic ultrasound on January 14, 2025 revealed bilateral ovarian lesions: a 1.6×1.1 cm mildly hyperechoic left mass and a 3.4×3.4×2.9 cm hyperechoic right mass. In accordance with current paraneoplastic neurological syndrome management guidelines, the patient underwent bilateral teratoma resection on January 15, 2025, consisting of right salpingo-oophorectomy with left ovarian cystectomy (ovary and fallopian tube preserved). Intraoperative frozen section confirmed benign mature teratomas bilaterally. Concurrently, the patient received intravenous immunoglobulin (IVIG) therapy at 25g daily for 5 consecutive days (January 13-17, 2025). Postoperatively, methylprednisolone pulse therapy was initiated on January 16 (1000mg daily) with subsequent tapering by 50% every 72 hours, reaching a maintenance dose of 60mg/day by January 30.During first-line immunotherapy, the patient’s consciousness showed marginal improvement; however, persistent delirium necessitated continuous sedation. Repeated EEG performed from January 26 to 27, 2025, demonstrated a persistent background dominated by fast-wave activity. Bilateral temporal regions exhibited increased theta slow-wave activity. No epileptiform discharges or rhythmic evolution were detected. During the examination, the patient displayed rapid, small-amplitude jerks involving the hands and oral region, accompanied by upward eye deviation. Notably, the jerking could be partially suppressed voluntarily for brief periods following commands. These findings are indicative of acute cortical dysfunction and are consistent with encephalitis-related EEG manifestations. Follow-up abdominal CT (01/23/2025) revealed colonic fecal loading with developing ileus. Concurrent persistent pyrexia precluded timely initiation of second-line therapy, requiring repeated antimicrobial administration and enema interventions. By February 8, 2025, with stabilized intermittent delirium and controlled infection, OFA therapy was commenced. Repeat lumbar puncture on February 11, 2025 demonstrated persistent anti-NMDAR IgG in CSF (titer 1:10) and serum (1:32), with residual anti-GFAP antibodies in CSF only (1:3.2). Clinical assessment revealed improved delirium by February 12 (MMSE: 20/30, moderate cognitive impairment),new neurological deficits: cephalalgia, bilateral lower limb weakness, and hypertonia, electrophysiological evidence of peroneal nerve dysfunction (reduced motor amplitudes on February 18 nerve conduction studies).Within one week of treatment, the patient demonstrated improvement in psychomotor agitation and involuntary vocalizations, though persistent cephalgia and new-onset autonomic dysfunction (nocturia, night sweats) were noted, with an mRS score of 3. While OFA re-administration was planned, serum B-cell enumeration (CD19+) on February 17, 2025 revealed complete depletion (0 cells/μL), confirming profound immunosuppression from prior anti-CD20 therapy. Given the profound B-cell depletion (CD19+ = 0/μL) and consequent high risk of systemic infections (particularly pulmonary) with additional OFA therapy, we transitioned to efgartigimod alfa - a novel FcRn-targeting biologic that selectively accelerates clearance of pathogenic IgG antibodies while preserving cellular immunity. The patient received intravenous infusion of efgartigimod alfa (400 μg) on February 17, 2025. By February 18, 2025, the patient demonstrated significant improvement in headache symptoms and marked alleviation of autonomic nervous dysfunction symptoms including nocturia. The modified Rankin Scale (mRS) score was 2 at this time. The patient demonstrated marked clinical response to efgartigimod alfa immunotherapy and was subsequently discharged on February 18, 2025 for continued rehabilitation and follow-up treatment at a local hospital. Post-discharge immunotherapy regimen included oral corticosteroids and mycophenolate mofetil. The patient was readmitted on March 11, 2025 due to the onset of limb myoclonus without identifiable precipitating factors. Diagnostic workup on March 12, 2025 revealed:CSF anti-NMDAR IgG (1:10 titer) and serum anti-NMDAR IgG (1:32 titer), with negative anti-GFAP antibodies in both serum and CSF;EMG showed reduced motor amplitude in bilateral peroneal nerves, with prolonged latency in the right peroneal nerve. Follow-up magnetic resonance imaging (MRI) of the head revealed no significant leptomeningeal enhancement or thickening, and no substantial subcortical lesions or abnormalities in morphology or size, indicating structural improvement on MRI (**Figure 1**). Repeat electroencephalography (EEG) demonstrated a background rhythm of 10 Hz (spectral range: 9–11 Hz), with theta slow waves in the bilateral temporal regions. Sharp waves and sharp and slow wave complexes were observed over the left temporal region, consistent with epileptiform discharges. The EEG findings remained consistent with anti-NMDAR encephalitis. Given the absence of clinical seizures, antiepileptic drug therapy was not initiated; instead, the patient was closely monitored for clinical changes. On March 14, 2025, repeat serum B-cell quantification (CD19+) remained undetectable. After excluding infectious contraindications, the patient received two additional intravenous infusions of efgartigimod alfa (800 μg) on March 14 and March 21, 2025.Following efgartigimod alfa immunotherapy, the patient exhibited clinical improvement and was subsequently discharged on March 21, 2025.

### 2.3 Follow-up

**Figure 2:**
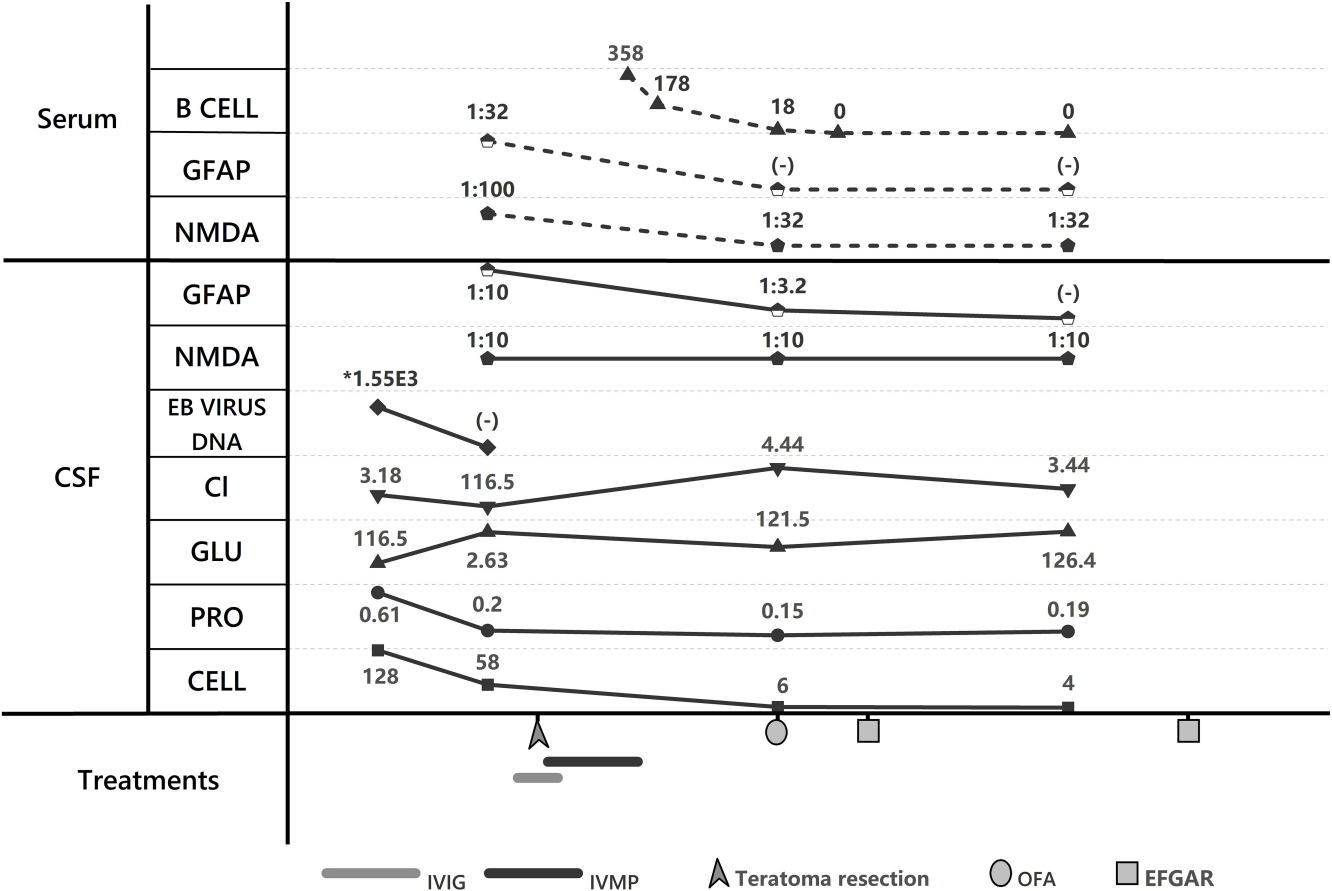
Laboratory trends and treatment course. GFAP, GFAP-IgG; NMDA, NMDAR-IgG; Cl, Chloride; GLU, CSF glucose; PRO, proteins; CELL, cell count; IVIG, IntraVenous Immunoglobulin; IVMP, IntraVenous Methylprednisolone; EFGAR, efgartimod; OFA, ofatumumab.

**Figure 3:**
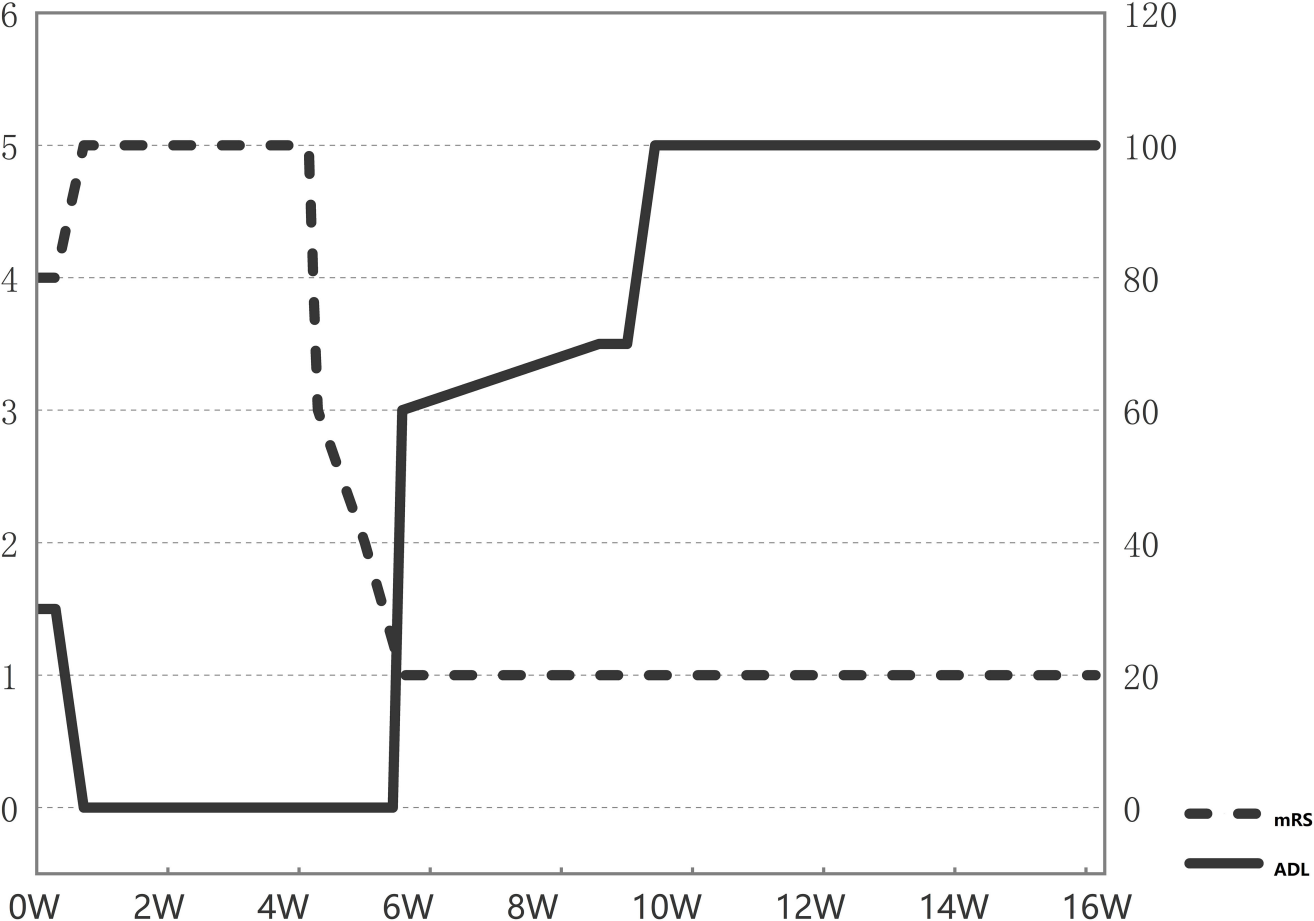
Serial changes in key patient scores. mRS, Modified Rankin Scale score; ADL, Activities of Daily Living score.

At the outpatient follow-up on April 21, 2025, the patient had been clinically stable for one month, with only residual bilateral lower limb weakness and no motor deficits. Electromyography revealed prolonged motor conduction latency and reduced amplitude in bilateral peroneal nerves; electroencephalography showed predominant low-amplitude fast-wave background activity with poor modulation, occasional sharp waves in left anterior temporal and left sphenoidal regions, and bilateral synchronous slow waves. The patient remained free of clinical seizures, and antiepileptic drug therapy was withheld. The persistent EEG abnormalities were considered attributable to acute cortical injury secondary to encephalitis, necessitating continued immunotherapy. At this stage, the modified Rankin Scale (mRS) score was 1 (**Figures 2**, **3**).

## 3 Discussion

### 3.1 Literature comparison supports the superiority of the sequential strategy

We searched PubMed for case reports on “OFA” and “Efgartimod” in treating autoimmune encephalitis (AE), excluding cases with: (1) single antibody positivity, (2) unclear clinical descriptions or follow-up, and (3) undocumented mRS scores. Two qualifying articles (14, 15) reporting 3 patients (including our current case) were identified. The clinical characteristics of these 3 patients are summarized (**Table 1**). All presented with anti-NMDAR antibody-positive encephalitis overlapping with other neural antibodies: one with concurrent anti-MOG antibodies, one with anti-LGI1 antibodies, and our case with anti-GFAP antibodies. Our patient demonstrated the shortest disease course, as the co-existing antibodies differed among cases (anti-MOG/LGI1/GFAP), we cannot exclude antibody-specific effects on disease progression. Both mRS improvement and recovery rate suggest that dual therapy (OFA plus efgartimod) shows non-inferior efficacy to monotherapy in double-antibody-positive cases, with superior safety profile compared to OFA monotherapy.

**Table 1:** Comparison of baseline characteristics among the three cases.

Case	Age/Sex	Type	PMH	Tumor	Efgar/OFA	Other treatments	Disease duration	Peak mRS	Best mRS
1	32/M	NMDALGI1	NO	NO	3efgar	MP,IVIG	120	5	1
2	49/M	NMDAMOG	type2 diabetes	NO	4ofa	MP,IVIG	79	5	2
3	24/F	NMDAGFAP	NO	teratoma	1ofa, 3efgar	MP,IVIG	74	5	1

PMH, Past Medical History; Efgar, efgartimod; OFA, ofatumumab; MP, Methylprednisolone; IVIG, IntraVenous Immunoglobulin.

### 3.2 Clinical significance and challenges of multiple antibody-positive AE

The present case initially developed viral encephalitis with symptom recurrence after initial treatment improvement. The patient underwent multiple referrals with progressive clinical deterioration without definitive diagnosis until being transferred to our department. Although definitive diagnosis was eventually established, multiple complications and risk factors delayed the initiation of both first-line and second-line immunotherapies. The patient demonstrated continuous clinical deterioration until receiving escalated immunotherapy with OFA. This represents the first reported case of autoimmune encephalitis complicated by dual seropositivity for anti-NMDAR and anti-GFAP antibodies alongside multiple comorbidities. Sequential therapy with OFA followed by efgartigimod was administered, resulting in marked clinical improvement without treatment-related adverse events. In cases of autoimmune encephalitis, the phenomenon of multiple antibody positivity is exceedingly rare, with less than 10% of cases demonstrating multiple antibody positivity (16). Different antibodies lead to distinct clinical manifestations and prognostic outcomes (17). NMDAR-AE primarily affects children and young women, frequently associated with ovarian teratomas, and typically presents with psychiatric symptoms, movement disorders, altered consciousness, autonomic dysfunction, seizures, and central hypoventilation (18). GFAP, involved in numerous biological functions of astrocytes, mainly affects the meninges, brain, spinal cord, and optic nerves, with clinical manifestations including fever, headache, encephalitis, myelitis, and optic neuritis (6). Some studies suggest that while anti-GFAP antibodies are associated with autoimmune encephalitis, they target intracellular components of glial proteins and may not be pathogenic themselves (19). The patient’s primary clinical manifestations included prodromal fever, delirium with psychiatric and behavioral abnormalities, autonomic dysfunction, and cognitive impairment (MMSE score: 20/30, baseline education: junior high school level), consistent with typical presentations of autoimmune encephalitis. Based on the prodromal fever and positive viral testing, the coexistence of anti-NMDAR and anti-GFAP antibodies in this case suggests a pathogenic interplay. This broader immune dysregulation, potentially triggered by a preceding viral infection combined with a teratoma, may underlie the dual seropositivity, possibly reflecting epitope spreading or polyclonal B-cell activation. These mechanisms likely contributed to the complex and refractory clinical course.

### 3.3 Discussion on etiology and predisposing factors

Tumors and viral encephalitis are recognized potential triggers for autoimmune encephalitis (1); notably, the present case presented with both conditions. A notable feature in this patient was the concomitant detection of Epstein-Barr virus (EBV) and human herpesvirus 7 (HHV-7) in the cerebrospinal fluid (CSF) during the initial phase of viral infection. A study profiling viruses and antibodies in the cerebrospinal fluid (CSF) of encephalitis patients detected Epstein-Barr virus (EBV) and human herpesvirus 6 (HHV-6) in a significant proportion of individuals with autoimmune encephalitis (20). HHV-7 is less frequently reported in studies of autoimmune encephalitis and is more commonly associated with children and adolescents (21). Consequently, the investigators considered Epstein-Barr virus (EBV) to be the primary viral trigger in this patient.

Teratoma, a germ cell tumor commonly found in young females, demonstrates a high prevalence among women with anti-NMDAR encephalitis (22). Current understanding proposes that the mechanism linking teratoma to autoimmune encephalitis involves the ectopic expression of NMDARs within neural elements of the teratoma, leading to a breakdown of immune tolerance; subsequently, the generated autoantibodies bind to receptors in the brain, ultimately resulting in encephalitis (23). Research proposes that ovarian teratomas can activate B cells to produce NR1-IgG and initiate a germinal center reaction. If teratoma removal is delayed, persistent antigen exposure may induce increased antibody affinity and generate long-lived plasma cells (LLPCs), rendering the patient refractory to teratoma resection and unresponsive to immunotherapy (24). The present case featured both viral infection and a teratoma. Following symptomatic improvement after treatment for viral meningitis initiated in response to pre-onset high fever, viral infection was considered the primary trigger. However, the coexisting teratoma represented an additional contributing pathogenic factor. Prompt screening and resection of the teratoma, albeit causing a delay in initiating first-line immunotherapy, ultimately facilitated subsequent therapeutic escalation, which achieved significant clinical efficacy.

### 3.4 Assessment of efficacy and safety of second-line therapy

Given that the clinical effects of RTX as a second-line agent typically manifest within 4 to 8 weeks—a relatively slow onset—and are associated with significant systemic side effects, the critical therapeutic time window in this complex autoimmune encephalitis case necessitated escalation of immunotherapy to prevent potential disease progression. OFA, a fully human type II anti-CD20 monoclonal antibody, confers advantages over RTX in terms of lower immunogenicity, thereby minimizing the development of anti-drug antibodies (ADAs) and reducing the incidence of hypersensitivity reactions. Furthermore, OFA exhibits a distinct biological profile by specifically targeting the juxtamembrane small extracellular loop epitope of CD20—a structural contrast to type I antibodies like RTX, which bind the distal membrane epitope. This unique binding mode enables OFA to lyse CD20-expressing cells resistant to RTX (25). OFA has emerged as a prominent research focus in autoimmune encephalitis in recent years. Its efficacy and safety have been validated across multiple studies. Particularly for complex and refractory autoimmune encephalitis, several case reports have documented its use as an alternative second-line therapy, enabling these patients to achieve significant efficacy with no adverse reactions (15, 25–28). Recently, a prospective multicenter cohort study on NMDAR encephalitis demonstrated that OFA provided significant efficacy and a favorable safety profile for patients. Notably, in patients who failed first-line immunotherapy, OFA treatment resulted in functional improvement with no reported serious adverse events (29).

Although OFA demonstrates a generally favorable safety profile, the FDA adverse event reporting series indicates that adverse reactions still occur. Among these, infections and infestations ranked as the second most common category of adverse events, preceded only by neurological disorders (30). The mechanism underlying its association with infections may be related to a reduction in circulating B-cell counts. Efgartigimod is a novel FcRn-targeting agent that accelerates the degradation of pathogenic antibodies. Compared to direct immunosuppression, efgartigimod offers greater specificity, rapidly reducing antibody titers while minimizing systemic side effects. In this case, sequential therapy with efgartigimod was selected due to the patient’s circulating B-cell count having reached zero, which was considered to confer a substantially elevated risk of infection. Although no current guidelines or expert consensus endorse its use for autoimmune encephalitis, clinical studies and case reports have demonstrated that efgartigimod can significantly alleviate symptoms and reduce the incidence of complications in this condition (14, 31–33). Its mechanism involves inhibiting the IgG recycling pathway, selectively accelerating the catabolism of pathogenic IgG without affecting other immunoglobulins or immune cells (34). This provides a complementary therapeutic approach to B-cell-depleting therapy with OFA. Following rapid B-cell depletion induced by OFA, the sequential initiation of efgartigimod facilitated accelerated antibody clearance. This combined approach mitigated risks associated with prolonged B-cell depletion while promoting symptom alleviation and disease stability. This represents a safe and manageable therapeutic strategy, offering a novel paradigm for managing complex and refractory autoimmune encephalitis.

## 4 Conclusion

For this patient with complex, refractory autoimmune encephalitis, presenting with multiple comorbidities and double-positive for autoantibodies, we initiated first-line therapy followed by an innovative escalation to sequential immunotherapy comprising OFA and efgartigimod. This approach ensured therapeutic efficacy while substantially reducing the risk of complications, thereby enhancing safety. It may have also accelerated clinical recovery. However, due to the single-case nature of this report, large-scale, multicenter clinical trials are warranted to validate the efficacy and safety of this therapeutic strategy.

## Data Availability

The raw data supporting the conclusions of this article will be made available by the authors, without undue reservation.
